# Near Infrared Light‐Emitting Diodes Based on Colloidal InAs/ZnSe Core/Thick‐Shell Quantum Dots

**DOI:** 10.1002/advs.202400734

**Published:** 2024-04-15

**Authors:** Hossein Roshan, Dongxu Zhu, Davide Piccinotti, Jinfei Dai, Manuela De Franco, Matteo Barelli, Mirko Prato, Luca De Trizio, Liberato Manna, Francesco Di Stasio

**Affiliations:** ^1^ Photonic Nanomaterials Istituto Italiano di Tecnologia Via Morego 30 Genova 16163 Italy; ^2^ Nanochemistry Istituto Italiano di Tecnologia Via Morego 30 Genova 16163 Italy; ^3^ Key Laboratory for Physical Electronics and Devices of the Ministry of Education & Shaanxi Key Lab of Information Photonic Technique School of Electronic Science and Engineering Xi'an Jiaotong University Xi'an 710049 China; ^4^ Dipartimento di Chimica e Chimica Industriale Università degli Studi di Genova Via Dodecaneso 31 Genova 16146 Italy; ^5^ Materials Characterization Facility Istituto Italiano di Tecnologia Via Morego 30 Genova 16163 Italy; ^6^ Chemistry Facility Istituto Italiano di Tecnologia Via Morego 30 Genova 16163 Italy

**Keywords:** Core/shell, InAs, Light‐emitting diodes, Near‐infrared, Quantum Dots, Restriction of hazardous substances (RoHS) compliant

## Abstract

Heavy‐metal‐free III–V colloidal quantum dots (QDs) exhibit promising attributes for application in optoelectronics. Among them, InAs QDs are demonstrating excellent optical performance with respect to absorption and emission in the near‐infrared spectral domain. Recently, InAs QDs attained a substantial improvement in photoluminescence quantum yield, achieving 70% at a wavelength of 900 nm through the strategic overgrowth of a thick ZnSe shell atop the InAs core. In the present study, light‐emitting diodes (LEDs) based on this type of InAs/ZnSe QDs are fabricated, reaching an external quantum efficiency (EQE) of 13.3%, a turn‐on voltage of 1.5V, and a maximum radiance of 12 Wsr^−1^m^−2^. Importantly, the LEDs exhibit an extensive emission dynamic range, characterized by a nearly linear correlation between emission intensity and current density, which can be attributed to the efficient passivation provided by the thick ZnSe shell. The obtained results are comparable to state‐of‐the‐art PbS QD LEDs. Furthermore, it should be stressed not only that the fabricated LEDs are fully RoHS‐compliant but also that the emitting InAs QDs are prepared via a synthetic route based on a non‐pyrophoric, cheap, and commercially available as precursor, namely tris(dimethylamino)‐arsine.

## Graphical abstract

Core/shell InAs/ZnSe Quantum Dots (QDs) synthesized via tris(dimethylamino)‐arsine route demonstrate remarkable emission properties. Light‐emitting diodes (LEDs) based on such QDs show emission at 896 nm with an external quantum efficiency of 13.3%, and maximum radiance of 12 Wsr^−1^m^−2^. Notably, the fabricated LEDs are compliant with the European directive on “Restrictions of Hazardous Substances in Electrical and Electronic Equipment” (RoHS).

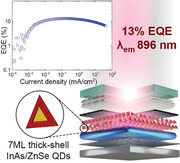

## 1 Introduction

In the field of optoelectronics, most efforts are dedicated toward improving device efficiency, sustainability, and stability.^[^ ^1^ ^]^ Multilayer solution processing is seen as a promising and cost‐effective approach to achieve the desired device performance, also enabling the use of low‐temperature and atmospheric pressure deposition methods like roll‐to‐roll coating, spray‐coating, spin‐coating, and inkjet printing.^[^ ^2^ ^]^ This strategy can reduce manufacturing costs and enhance the production of flexible, lightweight devices such as active matrix displays, sensors, and photovoltaic systems. For instance, displays are undergoing a transformative evolution, as witnessed by the emergence of organic light‐emitting diodes (OLEDs),^[^ ^3^ ^]^ quantum dot LEDs (QLEDs),^[^ ^4^ ^]^ and micro LEDs (µLEDs).^[^ ^5^ ^]^ Within the display industry, there is an ongoing endeavor to chart a technology roadmap that calls for innovative advancements in both form factor and functionality.^[^ ^6^ ^]^ OLEDs have demonstrated unparalleled efficiency within the visible spectral domain, thereby prompting extensive production of OLED displays accompanied by a remarkable commercial success and market demand. Contemporary OLED devices have attained an external quantum efficiency (EQE) over 25%.^[^ ^7^ ^]^

Regrettably, the EQE of OLEDs experiences a precipitous decline when approaching the near‐infrared (near‐IR, ranging from 800 to 2500 nm), plummeting to less than 4% around 900 nm wavelength in state‐of‐the‐art devices and even less than 0.1% for emission wavelengths longer than 1000 nm.^[^ ^8^ ^]^ In stark contrast, inorganic quantum dots (QDs) remain efficient emitters in the near‐IR and even in the mid‐IR (2500–25000 nm), where they showed efficient electroluminescence (EL) at 5000 nm.^[^ ^9^ ^]^ The most preeminent inorganic QDs exhibiting notable achievements in the IR spectral range are those based on lead sulfide (PbS). It is important to underscore the high photoluminescence quantum yield (PLQY) possessed by this material,^[^ ^10^ ^]^ particularly within the telecommunication spectral range (1300–1550nm).^[^ ^11^ ^]^ In a seminal work, Supran et al.^[^ ^12^ ^]^ employed PbS/CdS core/shell QDs obtaining a maximum EQE of 4.3% at 1242 nm. It was observed that the CdS shell played a pivotal role in attaining this remarkable EQE. On the other hand, Pradhan et al.^[^ ^13^ ^]^ developed IR QLEDs by employing a composite configuration comprising PbS QDs blended with ZnO nanoparticles as active layer. This approach yielded emission centered at 1400 nm and an EQE of 7.9%. In a recent work, they achieved an EQE of 11.8%.^[^ ^10^ ^]^ Another category of materials that emit in the IR are mercury chalcogenides.^[^ ^14^ ^]^ In this context, Qu et al. blended HgTe QDs and ZnO nanoparticles in the active layer,^[^ ^15^ ^]^ obtaining an EQE of 0.67% at 1300 nm, which has subsequently been improved to a 2.2% EQE.^[^ ^16^ ^]^ In the same spectral range, thanks to shell growth strategies, we have recently reached a maximum EQE of 7.5% using CdHgSe/CdZnS core/crown nanoplatelets.^[^ ^17^ ^]^

To date, the most efficient IR‐emitting QDs are not eligible for commercial applications since they are constituted by toxic elements, such as Cd, Pb, and Hg, that fall under the European restriction of hazardous substances (RoHS). Heavy‐metal‐free QDs such as silver chalcogenides,^[^ ^18^, ^19^ ^]^ CuInS_2_,^[^ ^20^, ^21^ ^]^ and InAs^[^ ^22^ ^]^ exhibit promising IR emission properties, indicating that an environmentally friendly alternative is feasible. CuInS_2_ and Ag_2_S have bulk bandgaps of 1.45^[^ ^23^ ^]^ and 1.1 eV,^[^ ^24^ ^]^ respectively, which restrict their spectral tunability to the near‐IR range, specifically between 700 and 1100 nm. In contrast, InAs is a III–V semiconductor with a narrow bulk bandgap of 0.35 eV^[^ ^25^ ^]^ and a large exciton Bohr radius (reported values of 45^[^ ^26^ ^]^ to 31.2 nm).^[^ ^27^ ^]^ This semiconductor has the potential of tuning the optical properties across a broad spectral range (from 700 nm up to 3500 nm),^[^ ^28^ ^]^ and it has already shown EL at 3300 nm wavelength in epitaxially grown systems.^[^ ^29^ ^]^ The potential for wide spectral tunability in the IR range makes InAs QDs a promising alternative to PbS, which offers the same spectral range tunability. However, numerous challenges have impeded utilization of InAs QDs in optoelectronic devices, for example: low PLQY and limited control over emission tunability due to challenges in increasing their size.^[^ ^30^, ^31^, ^32^ ^]^ Nonetheless, passivation of InAs QD surfaces by synthesizing core/shell structures is an effective approach for increasing the PLQY^[^ ^33^ ^]^ and enhancing the stability of the final QDs. A thick shell can effectively isolate the core region, resulting in significant enhancements in the performance of optoelectronic devices.^[^ ^34^, ^35^ ^]^ Consequently, thick shell QDs have elicited interest across a spectrum of optoelectronic applications, including solar cells,^[^ ^36^ ^]^ LEDs,^[^ ^37^ ^]^ hydrogen production,^[^ ^38^ ^]^ and lasers.^[^ ^39^ ^]^

The synthesis of InAs QDs is quite challenging and it has been traditionally carried out via highly reactive, pyrophoric, toxic, and costly chemicals, such as tris‐trimethylsilyl arsine (TMS‐As). TMS‐As based InAs QDs have been employed in QLEDs demonstrating EQEs of 4.6 and 13.3%, although these QDs are based on multi‐shell architectures also including InP and GaP, respectively.^[^ ^32^, ^40^ ^]^ With the aim of replacing TMS‐As with cheaper, less hazardous, and milder As precursors, in the last few years, several alternative compounds/reactants have been explored by the scientific community with tris(dimethylamino)‐arsine (amino‐As) having emerged as the most promising one.^[^ ^22^, ^41^, ^42^, ^43^, ^44^, ^45^, ^46^ ^]^ By employing amino‐As, Alane N,N‐dimethylethylamine as the reducing agent, and ZnCl_2_ as an additive, we recently formulated a synthetic approach for InAs QDs and their corresponding InAs/ZnSe core/shell QDs (shell thickness ≈1.5 monolayers, ML) demonstrating photoluminescence (PL) at 860 nm and a PLQY of 42% ± 4%.^[^ ^46^ ^]^ Employing such QDs we realized an LED exhibiting a relatively high turn‐on voltage of 2.7V, EQE of 5.5%, and maximum radiance of 0.2 Wsr^−1^cm^−2^.^[^ ^31^ ^]^ Following these initial results, we further improved our synthesis strategy obtaining InAs/ZnSe QDs with a tunable ZnSe shell thickness up to 7 ML, a remarkable PLQY of ≈70% ± 7% and PL peak at 900 nm in solution.^[^ ^47^ ^]^ In fact, in our previous work,^[^ ^47^ ^]^ we showed that the formation of an In–Zn–Se interlayer between the InAs core and the ZnSe shell contributed to reduce the strain at the core and shell interface. This interlayer, composed of In, Zn, Se, and cation vacancies, present a crystal structure similar to In_2_ZnSe_4_. The electronic structure of the thick‐shell QDs resembled that of type‐I heterostructures, further aiding in the more efficient confinement of excitons in the core region thanks to the ZnSe layer.

In this study, we focus on the design and fabrication of an LED with inverted architecture utilizing our 7 ML thick‐shell InAs/ZnSe QDs as an emissive layer. To date, there has been just one instance of a QLED crafted using InAs with a thin ZnSe synthesized by Amino‐As precursor.^[^ ^31^ ^]^ Now, this study marks the first development of a QLED employing InAs synthesized by Amino‐As with a thick ZnSe shell and enhanced performance. Our results show significant enhancement in LED performance compared to our previous findings,^[^ ^31^ ^]^ in terms of turn‐on voltage, maximum EQE, maximum radiance and dynamic range. Importantly, the champion LED reaches an EQE of 13.3% and radiance of 12 Wsr^−1^cm^−2^, figures‐of‐merit that are comparable to devices based on complex core/multi‐shell InAs QDs obtained via a tris‐trimethylsilyl (TMS) arsine route.^[^ ^32^ ^]^

## 2 Results and Discussion

**Figure 1:**
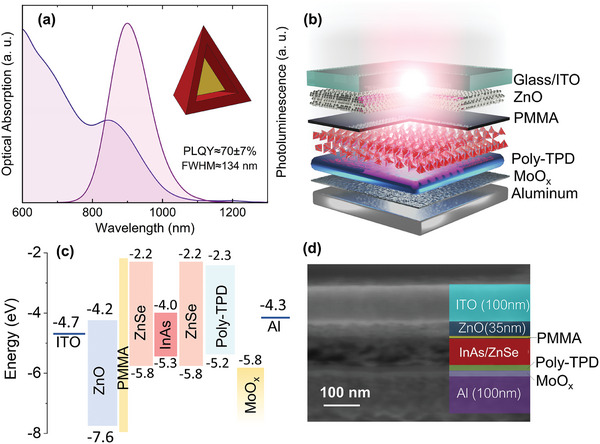
a) Photoluminescence and optical absorption spectra of the InAs/ZnSe QDs used in this study (inset: schematic of a single thick shell QD) b) the LED architecture, c) flat band energy diagram of the LED and d) scanning electron microscope (SEM) cross‐section image of a fabricated LED.

In the pursuit of designing an LED incorporating thick‐shell InAs/ZnSe QDs, we employ a hybrid structure, synergizing both inorganic and organic charge injection components. 7 ML thick‐shell InAs/ZnSe QDs were synthesized following our recently reported approach^[^ ^47^ ^]^ (see Experimental Section). The QDs having a size of ≈9 nm^[^ ^47^ ^]^ present a photoluminescence peak at 900 nm and a PLQY value of 70% ± 7% in solution and 55% ± 5% in solid film (see **Figure** **1**a for the optical properties of the QDs). Figure S1a (Supporting Information) illustrates the transmission electron microscope (TEM) image, and the inset of Figure 1a shows a scheme of the tetrahedral thick‐shell QDs used in our LEDs, whose structure is shown in Figure 1b. The LEDs were fabricated starting with an electron transport layer (ETL) based on ZnO,^[^ ^48^ ^]^ and a poly(methyl methacrylate) (PMMA) thin‐film; the latter serves as a modifier, allowing for precise control over the electron injection. This adjustment helps to attenuate the electron supply, promoting a more balanced charge distribution between the quantity of injected electrons and holes within the active layer. On the other hand, the hole transport layer (HTL) is based on Poly(4‐butylphenyl diphenylamine) (Poly‐TPD), which features the best band alignment (leading to a decreased turn‐on voltage) and best charge balance in the active layer (thus increasing the EQE). Indeed, in all previously reported InAs QLEDs, poly‐TPD was used as the HTL as well.^[^ ^31^, ^32^, ^40^ ^]^

Figure 1c presents a flat band energy diagram of the various layers constituting the LED. Thanks to its substantial bandgap energy (3.4 eV), ZnO remains optically inert within the infrared spectrum, thus obviating any optical absorption concerns. The valence band maximum (VBM) of the ZnO layer prepared with a sol–gel method was measured via ultraviolet photoemission spectroscopy (UPS, Figure S2b, Supporting Information), while the optical bandgap (and consequently the position of the conduction band minimum, CBM) was derived from the Tauc plot of the optical absorption edge. Figure S2a (Supporting Information) shows the corresponding Tauc plot for ZnO film with a 3.4 eV bandgap and the UPS analysis indicates the VBM position at −7.6 eV from vacuum. Regarding the InAs/ZnSe QDs, owing to the increased thickness of the ZnSe shell ranging from 3 to 4 nm,^[^ ^47^ ^]^ the UPS signal was exclusively obtained from ZnSe, as illustrated in Figure S1b (Supporting Information). This leads to a VBM energy of −5.8 relative to vacuum (refer to the Experimental Section for comprehensive details). The flat energy band values of InAs and the CBM position of ZnSe with respect to VBM utilized in this study were derived from our prior published manuscript,^[^ ^31^ ^]^ and relevant ref. [49]. Figure 1d shows the scanning electron microscope (SEM) cross‐section of a typical LED, where the thickness of the layers is visible: ITO (100 nm), ZnO (35 nm), PMMA (5 nm), the active layer (70 nm), and Poly‐TPD (15 nm), MoO_x_ (15 nm) and Aluminum (100 nm).

**Figure 2:**
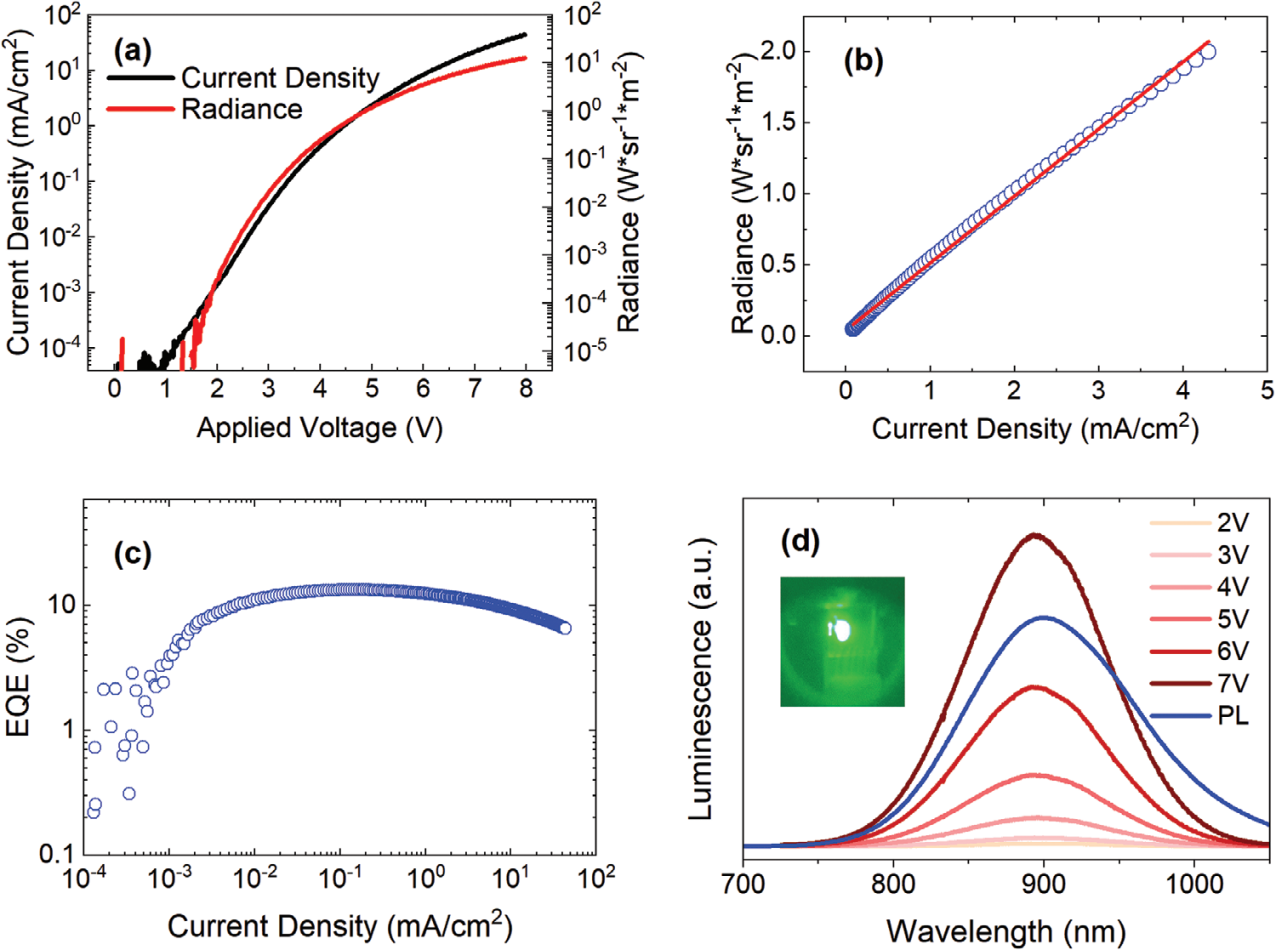
a) Radiance and current density of champion LED versus applied bias. b) Emission intensity (radiance) of champion device versus current density. c) external quantum efficiency of champion device versus current density. d) Electroluminescence spectrum at increasing applied bias and photoluminescence spectrum.

**Figure** **2**a shows the current density and radiance characteristics of the champion LED (i.e., LED with the highest EQE) as a function of applied bias. The turn‐on voltage of the device is around 1.5 V (estimated at a radiance value of 10^−5^ Wsr^−1^m^−2^), close to the bandgap energy of the emissive QDs at 900 nm (1.37 eV). Notably, the champion LED exhibits a maximum radiance of 12 Wsr^−1^m^−2^ at 8V.^[^ ^10^ ^]^ Moreover, within the dynamic range of 0.1 to 1.9 Wsr^−1^m^−2^, it displays an almost linear behavior with an R‐squared value of 0.998 for the fitted line in Figure 2b, making it particularly suitable for analog light modulation applications.^[^ ^50^ ^]^

The EQE curve of the champion LED is depicted in Figure 2c and it shows a maximum at 13.3%, corresponding to a current density of 1 × 10^−1^ mA.cm^−2^, a bias voltage of 3.49V, and a radiance of 8 × 10^−2^ Wsr^−1^m^−2^. Lastly, Figure 2d compares the EL under various bias voltages and the PL spectrum of a film of InAs/ZnSe core/shell QDs (an infrared camera image of the emitting LED is presented in the inset of Figure 2d). The EL spectrum is centered at 896 nm with a full‐width‐at‐half‐maximum (FWHM) of 112 nm. The EL shows a slight blueshift with a decreased FWHM compared to the PL of the QD film (PL peak at 900 nm, FWHM of 135 nm); yet, the spectra have been collected with two different spectrometers (see Experimental Section), thus the observed variations can be due to changes in sensitivity of the equipment employed, as much as other optical effects.^[^ ^51^, ^52^ ^]^ Furthermore, there is an absence of any prominent variation in spectral shape upon increasing the applied bias. This observation serves as evidence to indicate the lack of emissions originating from states associated with deep‐level traps.^[^ ^37^ ^]^

**Figure 3:**
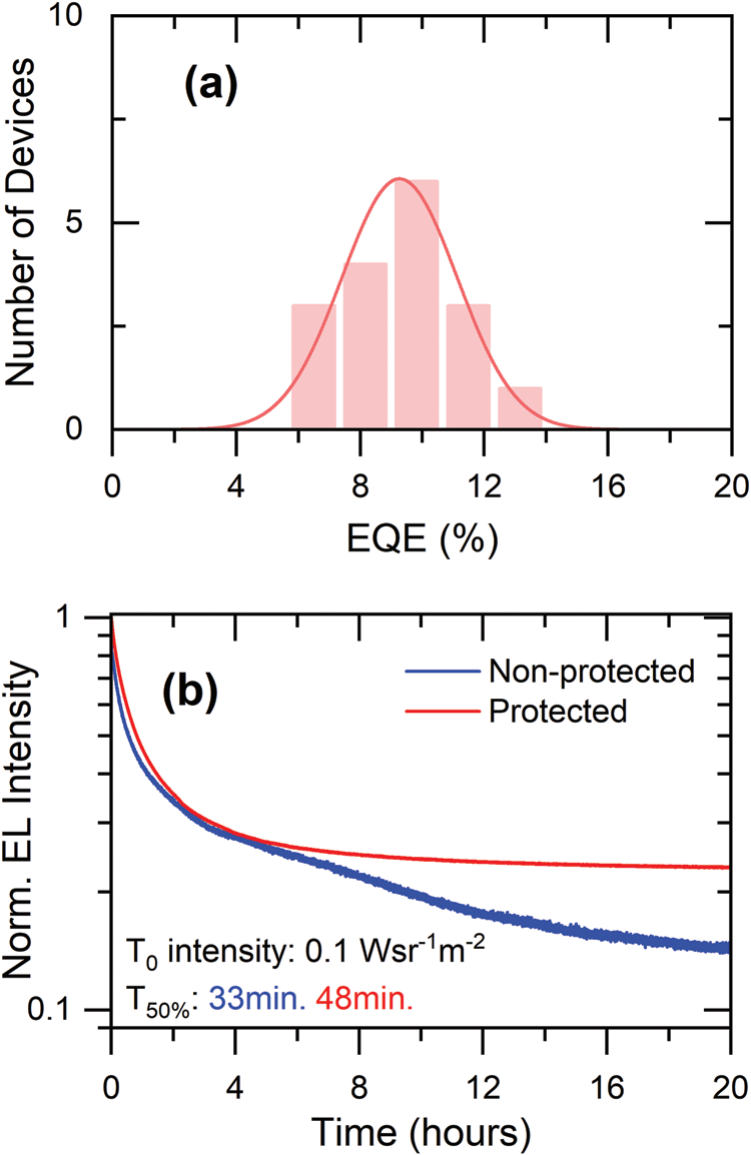
a) Histogram of *EQE*_max_ obtained from several fabricated devices b) stability test of QLEDs with and without protection layers in air.

In **Figure** **3**a, we report the maximum EQE of different LEDs with the same structure as the champion device. An average EQE of 10% has been achieved, reinforcing the reproducibility of the device fabrication presented in this study. All LEDs showed EQE values in the range of 5.7–13.3%. Furthermore, we evaluated the stability of our LEDs through continuous monitoring of the emission intensity under a constant bias in ambient conditions (Figure 3b). To enhance the device's stability in ambient air, we applied three layers of PMMA, PVA, and PVDF, inspired by Jagtap et al.,^[^ ^53^ ^]^ via spin‐coating over the LED (details provided in the Experimental Section). The initial light intensity was set at 0.1 Wsr^−1^m^−2^ corresponding to a current density of 0.2 mA cm^−2^. In the case of an unprotected device, it took 33 min for the light intensity to decrease to 50% of its initial value, which is commonly referred as T_50_. In contrast, the protected device exhibited a longer functional lifetime, lasting for 48 min before reaching T_50_. The protected device's emission intensity reduces to 30% of the initial value after ≈4 h and from that point, the intensity becomes stable for a considerable time. QD LEDs suffer from short stability of luminescence in comparison to OLEDs used in consumer electronics.

It is insightful to compare the results from our core‐thick shell QLEDs with other LEDs with the same device architecture but other types of InAs QDs having different PLQYs (although obtained with the same synthetic approach based on amino‐As). In fact, such comparison can help us understand if the improved performance of our QLEDs, compared to those of the devices we previously fabricated with 1.5 ML shell QDs, is only related to the improved PLQY of thick‐shell InAs/ZnSe QDs or if it is also due to a different electrical behavior.

We already reported LEDs made with 1.5 ML shell InAs/ZnSe QDs; yet,^[^ ^31^ ^]^ with a different structure than the inverted one used here. Therefore, we tested in the present work the 1.5 ML shell InAs/ZnSe QDs and InAs core only QDs in the same inverted LED architecture used for the champion device. The PL and absorption spectra of core only InAs QDs and 1.5 ML shell InAs/ZnSe QDs are shown in Figure S3 (Supporting Information). Core only QDs are barely emitting at 890 nm with a PLQY ≈1% ± 0.1%; on the other hand, the 1.5 ML shell InAs/ZnSe QDs show emission centered at 978 nm with a PLQY of 36% ± 4%. Additionally, we investigated how varying the thickness of the shell impacts the PL stability of QD solutions (Figure S4, Supporting Information). After a storage for 154 days, PL measurements of the samples revealed that QDs with thicker shells (7 ML) exhibited higher stability in their emission. Specifically, the core‐only and the 1.5 ML QDs showed a PL red‐shift of 57 and 32 nm, respectively. On the other hand, the 7 ML QDs maintained an emission profile akin to freshly prepared samples. The performance of LEDs based on core only InAs and 1.5 ML shell InAs/ZnSe QDs are depicted in Figure S5 (Supporting Information). Both LEDs exhibit a turn‐on voltage slightly lower than the 7ML thick‐shell QLED, ≈1.4 V, which aligns closely with the bandgap energy of the emissive layer, and it can be assigned to the decreased thickness (or absence) of the ZnSe shell. The maximum bias voltage was selected to ensure that the current density did not exceed a safe condition for all three types of LEDs based on initial experiments. Accordingly, the maximum bias voltage was chosen at 4.5 V for core InAs QDs, 7 V for 1.5 ML shell QDs, and 8 V for 7 ML thick‐shell QDs. Figure S5e (Supporting Information) illustrates the EQE curves of the three types of devices, where the *EQE*_max_ value for 1.5 ML shell QDs is 5.9% and core‐only InAs LED exhibits 0.029%. As the EQE of a QLED is directly proportional to the PLQY of the emitting QDs,^[^ ^54^ ^]^ a dramatic enhancement in EQE is achieved by substituting lowly emitting QDs with much brighter ones. In fact, QDs with 1.5 ML shell exhibit a PLQY of 36% and the respective LEDs achieve an EQE of 5.9%. Conversely, utilizing QDs with a 7 ML shell and a PLQY of 70%, the maximum EQE reaches 13.3%. This suggests that the EQE appears to be approximately five to six times lower than the PLQY value, thus corroborating the hypothesis that improved EQE can be mainly ascribed to the higher PLQY of the 7 ML thick‐shell InAs/ZnSe QDs in our device architecture, similar to what observed by Supran et al.^[^ ^12^ ^]^ in PbS core‐only and PbS/CdS core/shells. It is important to note that the superiority of the 7 ML thick‐shell InAs QLED extends beyond the *EQE*_max_ value. In contrast to the core‐only InAs and 1.5 ML shell InAs QLEDs, which both experience a significant one‐order‐of‐magnitude EQE roll‐off to the point of maximum radiance, the 7 ML thick‐shell QLED demonstrates a diminished EQE roll‐off, decreasing from 13.3% to 6.5%. The accumulation of excess electrons, leading to negatively charged QDs, is likely to suppress the efficiency of EL in each QD due to Auger recombination, particularly at high current density.^[^ ^55^ ^]^ The presence of thick 7 ML ZnSe shells can hinder electron transfer to the InAs core by acting as a more potent potential barrier between the latter and the ETL, diminishing QD charging even under elevated current densities. Consequently, QDs with thicker ZnSe shells exhibit enhanced EL efficiencies and less pronounced efficiency roll‐off across a broad current density range. In addition, our previous investigation on InAs/ZnSe QDs showed that, the thick ZnSe shell slightly mitigates the non‐radiative Auger recombination process in InAs cores.^[^ ^47^ ^]^

The radiance–current density (R–J) graph depicted in Figure S5f (Supporting Information) demonstrates a significant impact of ZnSe shell on required minimum current density to induce EL in our QLEDs. In fact, we observe a reduction from 0.1 to 0.0004 mA cm^−2^ when substituting the core only InAs QDs to the core/shell QDs. The maximum radiance obtained from core InAs QDs and 1.5 ML shell QLEDs is 0.02 and 3.6 Wsr^−1^m^−2^, respectively, significantly lower than that of the 7 ML thick‐shell InAs/ZnSe QLED. The 7 ML thick‐shell QLED not only demonstrates the highest radiance but also the greatest emitting dynamic range, starting from 4 × 10^−4^ to 42 mA.cm^2^ (12.5 Wsr^−1^m^−2^). Finally, the 7 ML thick‐shell InAs QLED exhibits the most robust emission stability among all devices (Figure S6, Supporting Information). This phenomenon can be attributed to the improved durability and decreased trap density imparted by the thick ZnSe shell (the latter demonstrated by the improved PLQY with respect to the other QDs). It is also possible that such a thick shell is an effective method for reducing the release of indium and arsenic components into the surroundings, making these QDs suitable for use in biological imaging, although a thorough investigation on their stability in various environments is required.

In summary, the integration of 7 ML thick‐shell InAs/ZnSe QDs in LEDs based on inorganic and organic charge transport layers has yielded remarkable devices with substantial radiance and commendable reproducibility. The device's performance is compared to state‐of‐the‐art near‐IR QLEDs in Table S1 (Supporting Information). The maximum EQE, radiance, and turn‐on voltage rank QLED among the top‐performing ones in the literature.

InAs QDs are a competitive substitute for infrared‐emitting semiconductors containing heavy metals. Nevertheless, challenges persist in realizing efficacious emissive InAs QDs at wavelengths above 1000 nm. Consequently, a protracted trajectory of research lies ahead to investigate innovative synthesis methodologies and fabrication of optoelectronic devices founded on InAs QDs emitting in the telecommunication window (1300–1600 nm).

## 3 Experimental Section

### Materials Used

The following materials were employed in this study: indium(III) chloride (denoted as InCl_3_, purity: 99.999%, sourced from Sigma–Aldrich), zinc(II) chloride (referred to as ZnCl_2_, purity: 99.999%, sourced from Sigma–Aldrich), zinc acetate dehydrate (sourced from Sigma–Aldrich), ethanolamine (sourced from Sigma–Aldrich), 2‐methoxyethanol (sourced from Sigma–Aldrich), Poly(4‐butyl‐N,N‐diphenylaniline) (referred to as Poly‐TPD, sourced from Ossila), polymethyl methacrylate (PMMA), Poly(vinyl alcohol) (PVA), Polyvinylidene fluoride (PVDF), Molybdenum oxide (referred to as MoO_3_, sourced from Alpha Chemicals), Chlorobenzene (sourced from Sigma–Aldrich), tris(dimethylamino)arsine (abbreviated as amino‐As, purity: 99%, sourced from Strem), alane N,N‐dimethylethylamine complex solution (referred to as DMEA–AlH_3_, in a 0.5 m solution in toluene, sourced from Sigma–Aldrich), selenium powder (denoted as Se, purity: 99.99%, sourced from Strem), oleylamine (referred to as OLAM, purity: 98%, sourced from Sigma–Aldrich), tri‐n‐octylphosphine (abbreviated as TOP, purity: 97%, sourced from Strem), toluene (anhydrous, purity: 99.8%, sourced from Sigma–Aldrich), ethanol (anhydrous, purity: 99.8%, sourced from Sigma–Aldrich), hexane (anhydrous, purity: 95%, sourced from Sigma–Aldrich), and N,N‐dimethylformamide (abbreviated as DMF, anhydrous, purity: 99.8%, sourced from Sigma–Aldrich). All chemicals were utilized without further purification.

### Preparation of Amino‐As Precursor

In a glovebox filled with nitrogen (N_2_), 0.2 mmol of amino‐As was dissolved in 0.5 mL of degassed oleylamine at 40 °C for 5 min until no further bubbles were observed.

### Preparation of 1 M TOP‐Se Precursor

In a nitrogen‐filled glovebox, 10 mmol of Se powder was combined with 10 mL of trioctylphosphine (TOP) in a 20 mL glass vial. The mixture was heated to 250 °C under constant stirring for ≈30 min to create a transparent solution, which was then cooled to room temperature.

### Preparation of 0.8 m ZnCl_2_‐OLAM Precursor

In a nitrogen‐filled glovebox, 8 mmol of ZnCl_2_ was mixed with 10 mL of oleylamine (OLAM) in a 20 mL glass vial. The mixture was heated at 250 °C under constant stirring for ≈50 min. Due to the solidification of the 0.8 m ZnCl_2_‐OLAM Precursor at room temperature, it was necessary to preheat it before transferring it into a syringe.

### Synthesis of InAs Core QDs

InAs core QDs were synthesized with minor modifications based on the previous work.^[^ ^46^, ^47^ ^]^ In a typical synthesis, 0.2 mmol of InCl_3_, 4 mmol of ZnCl_2_, and 5 mL of OLAM were loaded into a 100 mL three‐necked flask under an inert atmosphere. The mixture was degassed at room temperature for 10 min and then at 120 °C under vacuum for 40 min. The flask was heated to 180 °C under N_2_ to dissolve all precursors, then cooled to 120 °C and pumped under vacuum for an additional 30 min. The mixture was heated to 240 °C under nitrogen, and the amino‐As precursor was swiftly injected into the flask, followed by the injection of 1.2 mL of the DMEA‐AlH_3_ toluene solution. The temperature was rapidly increased to 300 °C (≈30 °C min^−1^), and the reaction was allowed to run for 15 min after which it was quenched by removing the flask from the heating mantle. The QDs were washed twice with toluene and ethanol, precipitated by centrifugation at 4000 rpm, and dispersed in toluene for further characterization.

### Synthesis of InAs/ZnSe Core/Shell QDs

The synthesis of the ZnSe shell was performed following the strategy reported in the previous work.^[^ ^47^ ^]^ In details, after quenching the growth of InAs QDs by cooling the reaction mixture down to 90 °C (by removing the flask from the heating mantle), 3.5 mL of 0.8 m ZnCl_2_‐OLAM was added to the crude reaction mixture, followed by the injection of 7.5 mL TOP‐Se. The resulting mixture was heated up to 310 °C (≈30 °C min^−1^). Samples with different shell thicknesses were obtained by quenching the shell growth at different reaction times: 0 min (1.5 ML) and 120 min (7 ML). The QDs were washed with toluene and ethanol, precipitated by centrifugation at 2000 rpm twice, and dispersed in toluene for further characterization.

### Optical Properties

The absorption spectra were measured using a Varian Cary 5000 UV−vis−NIR spectrophotometer. The samples were prepared by diluting QD samples in 3 mL of toluene within 1 cm path‐length quartz cuvettes. These cuvettes were closed with airtight screw caps and prepared in a glovebox filled with nitrogen (N_2_). Photoluminescence spectrum and quantum yield were conducted utilizing a Edinburgh Instruments FLS900 fluorescence spectrometer equipped with an integrating sphere, using the Xe lamp's output for excitation. Each QD solution underwent dilution to achieve an optical density of ≈0.1 at the excitation wavelength.

### Ultraviolet Photoelectron Spectroscopy (UPS)

UPS investigations were conducted using a Kratos Axis UltraDLD spectrometer equipped with a He I (21.22 eV) discharge lamp. The measurements were performed on a 55 µm diameter area, employing a pass energy of 10 eV and a dwell time of 100 ms. To precisely determine the low‐kinetic‐energy (i.e., high‐binding‐energy) cutoff, a −9.0 V bias was applied to the sample, and a take‐off angle of 0° with respect to the sample normal was used, following the methodology outlined by Helander et al.^[^ ^56^ ^]^ The estimation of the valence band maximum relative to the vacuum level involved measuring the energy difference between the high‐ and low‐binding energy cutoffs.^[^ ^57^ ^]^

Figure S1c (Supporting Information) presents UPS data acquired from InAs/ZnSe core/shell QDs with a 7 ML thick shell, utilized in the fabrication of LEDs. Following the methodology outlined in ref. [54], the VBM position relative to the vacuum level was estimated by analyzing the separation between the onsets of high and low‐binding energy, determined through linear fitting (illustrated by red segments in Figure S1c, Supporting Information). The calculated VBM position for InAs/ZnSe core/shell QDs with a 7 ML thick shell is −5.8 ± 0.2 eV. Leveraging the optical bandgap values of 1.3 eV for InAs and 3.5 eV for ZnSe (derived from Refs. [31, 58]), a comprehensive energy diagram for the QDs was constructed, as depicted in Figure 1c.

### TEM and SEM Measurements

Bright‐field TEM images of the QDs were acquired by a JEOL JEM‐1400 Plus transmission electron microscope equipped with thermionic source (LaB_6_) operating at an acceleration voltage of 120kV. The images were acquired by Gatan CCD camera Orius 830 (2048 × 2048 active pixels). The sample was prepared by drop‐casting onto carbon film‐coated 200 mesh copper grids a diluted QD dispersion. The cross‐section was performed via a FEI Helios NanoLab DualBeam 650 system (a scanning electron microscope/ focused ion beam workstation) with the Schottky FESEM column (Elstar) and the 30 kV Ga Focused Ion Beam (Tomahawk) placed at 52°one with respect to the other.

### LEDs Fabrication

The fabrication process was started using pre‐patterned ITO glasses and it involved the deposition of a ZnO layer through a sol–gel method. To start this process, a sol–gel solution was prepared by dissolving 1.6 g of zinc acetate dihydrate (Zn(CH_3_COO)_2_·2H_2_O) and incorporating 100 µL of ethanolamine into a volume of 5 mL of 2‐methoxyethanol. The solution underwent an overnight period of stirring to ensure homogeneity before utilization.

Subsequently, 30 µL of the prepared sol–gel precursor was spin‐coated at 4000 rpm for a duration of 50 s over the ITO glass substrate. Following this spinning procedure, the sample was annealed at 200 °C for 20 min. This annealing procedure was done in ambient air and after that, the samples were taken into a glovebox. A PMMA layer was spun at 2000 rpm using 1.5 mg mL^−1^ PMMA in acetone followed by 90 °C annealing. The QDs solution with a concentration of 30 mg mL^−1^ in toluene was used for spin coating the active layer. The active layer was spun at 2000 rpm for two times, followed by annealing at 70 °C after each spin coating process. Poly‐TPD with a concentration of 10 mg mL^−1^ in chlorobenzene was spun over the active layer at 2000 rpm followed by annealing at 70 °C. After the various spin coating steps, the samples were moved to the thermal evaporator inside the glovebox to deposit 15 nm MoO_3_ and 100nm aluminum in high vacuum (2 × 10^−6^ mbar).

### LEDs Encapsulation

In order to increase the air stability of the device, three encapsulation layers were spin coated over the aluminum contact. A protective layer comprising PMMA/PVA/PVDF was applied as follows: A PMMA solution (5 wt.% in CHCl_3_) was spin‐coated onto the substrates at 2000 rpm for 60 s, followed by a brief annealing step at 50 °C for 1 min. Subsequently, PVA (a centrifuged solution at 10 wt.% in water) and PVDF (10 wt.% in DMF) were spin‐coated at 4000 rpm for 60 s and 1500 rpm for 30 s, respectively. After each coating, the substrates underwent a 1 min annealing process at 50 °C. Finally, the device was placed in a vacuum overnight to allow for the complete drying of the encapsulation layers.

### LEDs Characterization

The present investigation involves the characterization of devices having an area of 4.5 mm^2^. This characterization includes the analysis of their current density and radiance concerning the applied bias. The measurements were carried out in ambient air with a relative humidity ≈50%. To apply the bias and measure the current passing through the device, a Keithley 2636 source‐measure unit was utilized. The emitted light was quantified using a calibrated Gentec germanium‐based photodetector (model PH20‐Ge‐D0). Radiance (*R*) was determined using the formula 𝑅 = $\frac{L_{0}}{S_{2}\;}$ (expressed in units of W sr^−1^ m^−2^), where S_2_ represents the emissive area, and L_0_ signifies the radiant intensity, denoting the power of light emitted from a source within a solid angle unit. Assuming that the source followed a Lambertian profile, the following equation: 𝐿_0_ = 𝑃 Ω = 𝑃 𝑙_2_/𝑠_1_ (in units of W sr^−1^) was employed. In this equation, P represents the emitted power in the forward direction perpendicular to the LED surface, and Ω = 𝑙_2_/*s*_1_ is the solid angle between the emitting area and the photodetector (where *l*_2_ indicates the distance between them, and *s*_1_ refers to the photodetector's area).

The EQE (External Quantum Efficiency) was determined as the ratio of the number of emitted photons (Np) in the forward direction in free space to the number of injected charge carriers (Ne): 𝐸𝑄𝐸 = 𝑁𝑝/𝑁𝑒. To calculate Np, the following formula was used:

(1) $$N_{p}=\frac{\pi \times P\times l_{2}\times \lambda }{S_{1}\times \mathrm{hc}}$$

where λ represents the emission wavelength, and hc is the product of the Planck constant (*h* = 6.62 × 10^−34^ Js) and the speed of light in vacuum (*c* = 3 × 10⁸ m s^−1^). The injected charge carrier 𝑁𝑒 was obtained as 𝑁𝑒 = 𝐼/𝑞, where *I* represents the current flowing into the device, and *q* is the elementary charge constant (1.6 × 10^−19^ C).

To evaluate the device's stability, a test in air with a constant applied voltage was conducted, measuring radiant power using the Ge photodetector continuously for 24 h. Electroluminescence spectrum measurements were carried out using an Acton750i Princeton spectrometer coupled with PIXIS (CCD) camera.

## Conflict of Interest

The authors declare no conflict of interest.

## Supporting information

Supporting Information

## Data Availability

The data that support the findings of this study are available from the corresponding author upon reasonable request.
