# Supplementary material for: Near Infrared Light‐Emitting Diodes Based on Colloidal InAs/ZnSe Core/Thick‐Shell Quantum Dots
Source: Adv Sci (Weinh). 2024 Apr 15;11(23):2400734. doi: 10.1002/advs.202400734 (PMC11187924; doi:10.1002/advs.202400734)
Supplement: Supplementary file 1 — Supporting Information [file ADVS-11-2400734-s001.pdf]

ADVANCED SCIENCE
Open Access

Supporting Information

for *Adv. Sci.*, DOI 10.1002/advs.202400734

Near Infrared Light-Emitting Diodes Based on Colloidal InAs/ZnSe Core/Thick-Shell Quantum Dots

*Hossein Roshan, Dongxu Zhu, Davide Piccinotti, Jinfei Dai, Manuela De Franco, Matteo Barelli, Mirko Prato, Luca De Trizio, Liberato Manna and Francesco Di Stasio**

Near infrared light-emitting diodes based on colloidal InAs/ZnSe core/thick-shell quantum dots

Hossein Roshan,[1] Dongxu Zhu,[2] Davide Piccinotti,[1] Jinfei Dai,[2,3] Manuela De Franco,[1,4] Matteo Barelli,[1] Mirko Prato,[5] Luca De Trizio,[6] Liberato Manna,[2] Francesco Di Stasio [1,*]

[1] Photonic Nanomaterials, Istituto Italiano di Tecnologia, Via Morego 30, 16163, Genova, Italy

[2] Nanochemistry, Istituto Italiano di Tecnologia, Via Morego 30, 16163 Genova, Italy

[3] Key Laboratory for Physical Electronics and Devices of the Ministry of Education & Shaanxi Key Lab of Information Photonic Technique, School of Electronic Science and Engineering, Xi'an Jiaotong University, Xi'an, 710049, China

[4] Dipartimento di Chimica e Chimica Industriale, Università degli Studi di Genova, Via Dodecaneso 31, 16146 Genova, Italy

[5] Materials Characterization Facility, Istituto Italiano di Tecnologia, Via Morego 30, 16163 Genova, Italy

[6] Chemistry Facility, Istituto Italiano di Tecnologia, Via Morego 30, 16163 Genova, Italy

Francesco Di Stasio: Francesco.distasio@iit.it

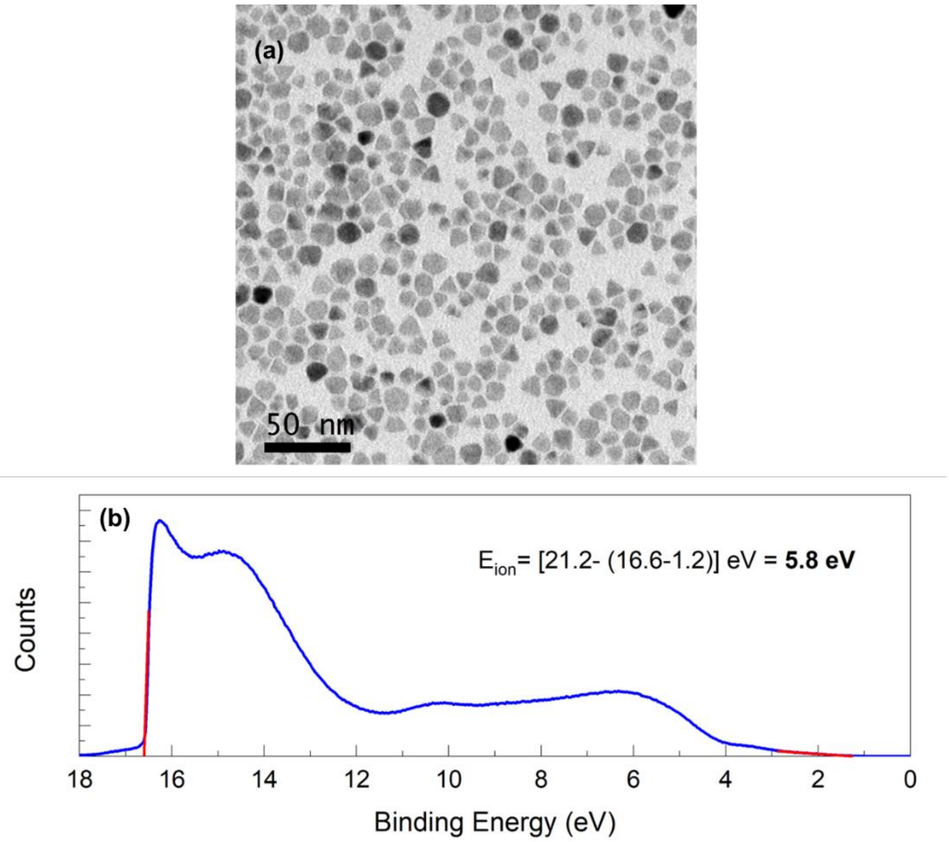

Figure S1. (a) TEM image of InAs/ZnSe core-thick shell QDs. (b) ultraviolet photoemission spectroscopy (UPS) analysis of a InAs/ZnSe core-thick shell QD film.

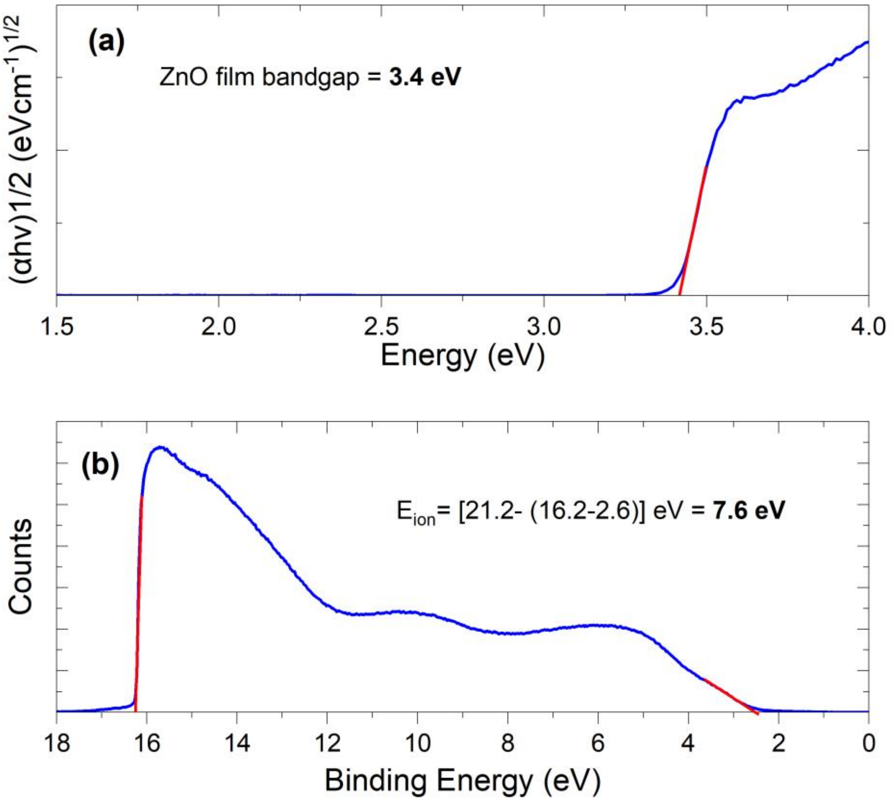

Figure S2. (a) Tauc plot and (b) UPS spectrum of a ZnO layer prepared via sol-gel method.

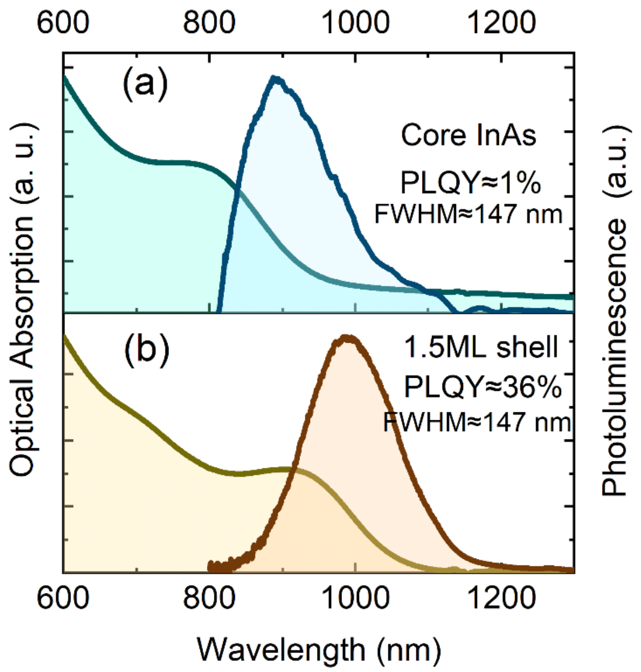

Figure S3. Optical absorption and photoluminescence spectra of (a) core-only QDs and (b) 1.5ML shell QDs in toluene.

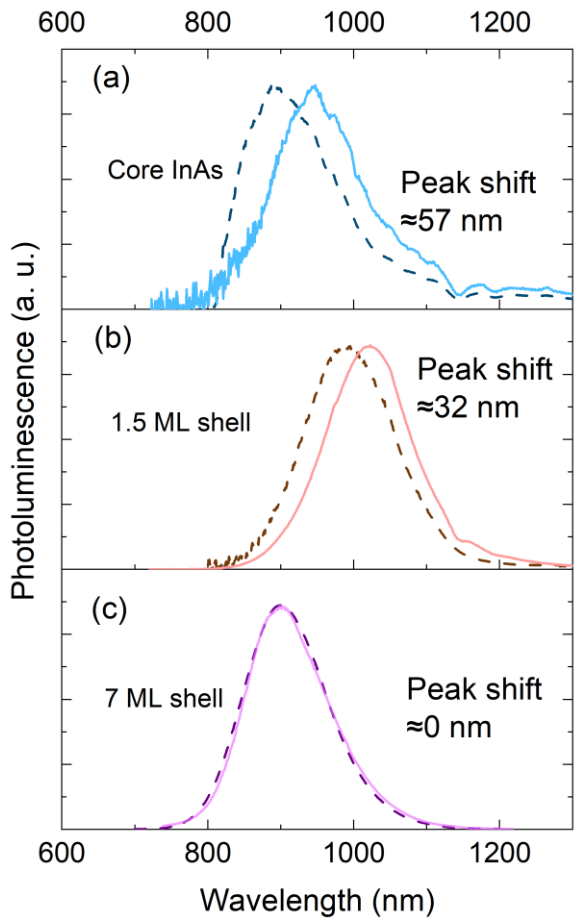

Figure S4. Photoluminescence spectra of (a) core-only QDs, (b) 1.5ML shell, and (c) 7 ML shell QDs in toluene measured from fresh-made samples (dashed lines), and after storage for 154 days (solid lines).

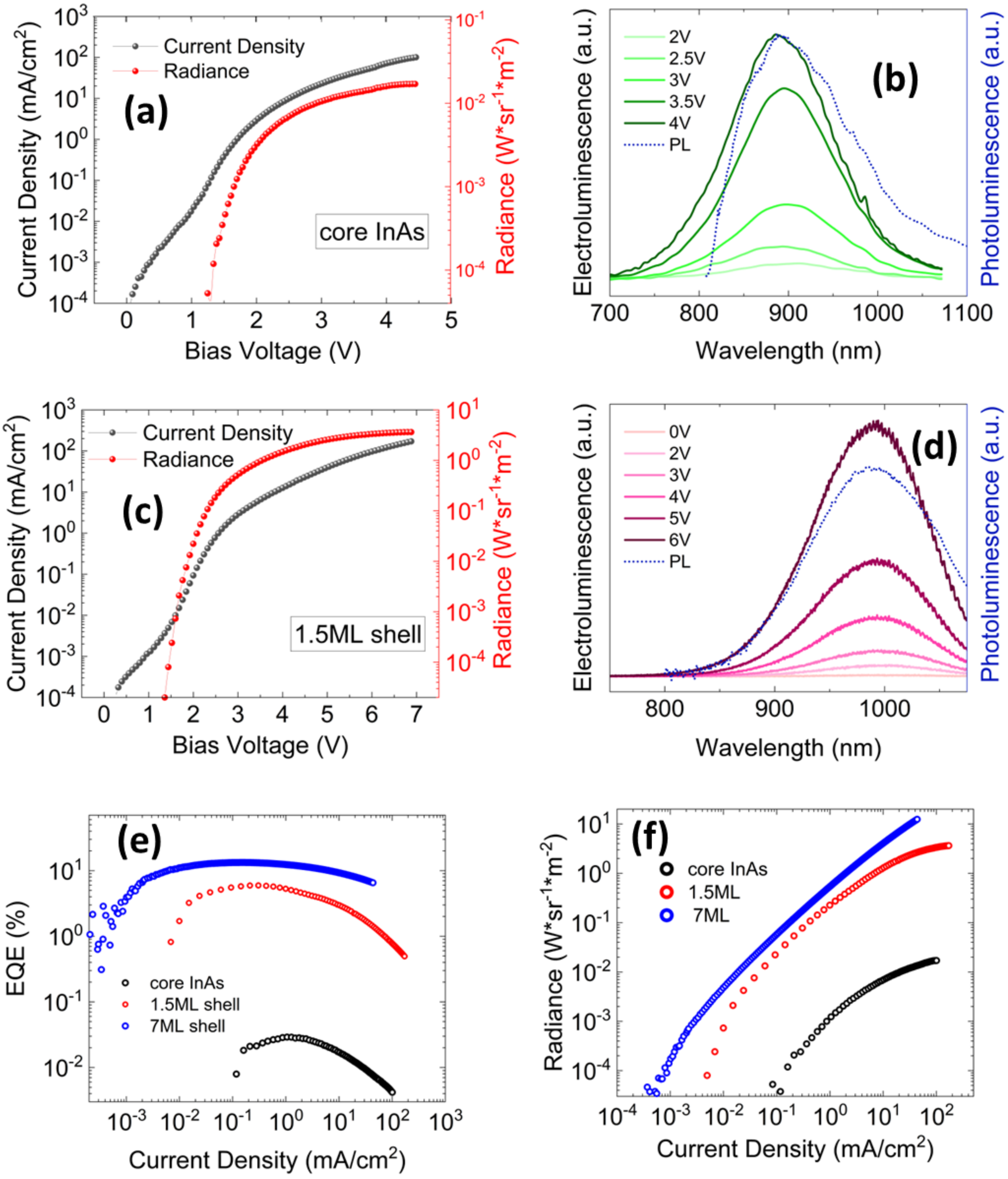

Figure S5. (a) current density and radiance of a core-only InAs QLED versus applied bias. (b) electroluminescence and photoluminescence of core-only InAs QDs. (c) current density and radiance of InAs/ZnSe 1.5ML QLED versus applied bias. (d) electroluminescence and photoluminescence of InAs/ZnSe 1.5ML QDs. (e) Comparison of EQE curves of core-only InAs,1.5ML and 7ML shelled InAs QDs. (f) Radiance versus current density curves of core InAs, 1.5ML and 7ML shelled InAs QDs.

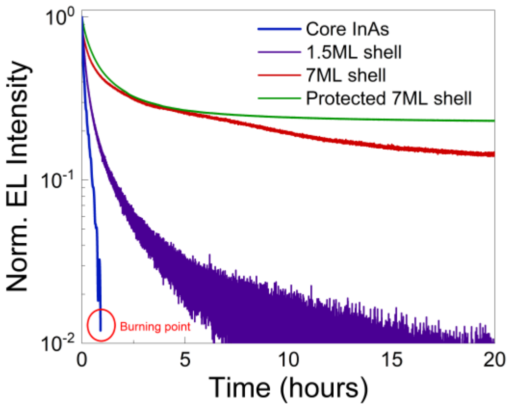

Figure S6. Stability test of different devices using core-only InAs, 1.5ML and 7ML shelled InAs QLEDs.

Table S1. Comparison table of state-of-the-art LEDs emitting in the 800~1000nm spectral range: type of active material employed, photoluminescence quantum yield of the active material (PLQY), type of electron transport layer (ETL), type of hole transport layer (HTL), turn-on voltage (V_{on}), maximum radiance (R_{max}), stability (St.), electroluminescence peak wavelength (EL), maximum external quantum efficiency (EQE_{max}), year of publication (year) and respective reference.

**Active material**	**PLQY (%)**	**ETL**	**HTL**	**V_{on} (V)**	**R_{max} ($W\ sr^{-1} m^{-2}$)**	**St.**	**EL (nm)**	**EQE_{max} (%)**	**year**	**Ref.**
conjugated polymer	-	Ca	PDOT:PSS	1.1	-	-	970	0.05	2004	[1]
PbS@CdS	20	TiO2	P3HT	1.47	-	-	900	0.002	2014	[2]
polymer	-	Ca	PDOT:PSS	14.8<br>28<br>23	-	-	890<br>927<br>1000	0.091<br>0.006<br>0.018	2015	[3]
$CsSnI_3$	-	PBD/LiF	PDOT:PSS	1.5-2	40	-	950	3.8	2016	[4]
$MASnI_3$	-	F8/Ca	PDOT:PSS	2	3.4	4m	945	0.72	2016	[5]
polymer	-	TPBi	PDOT:PSS	9	-	-	750	0.15	2018	[6]
FPMAI-$MAPb_{0.6}Sn_{0.4}I_{2.4}Br_{0.6}$<br>FPMAI-$MAPb_{0.6}Sn_{0.4}I_{2.6}Br_{0.4}$<br>FPMAI-MASnI3<br>FPMAI-$MAPb_{0.6}Sn_{0.4}I_{2.8}Br_{0.2}$<br>FPMAI-$MAPb_{0.6}Sn_{0.4}I_3$<br>FPMAI-$MAPb_{0.4}Sn_{0.6}I_3$<br>FPMAI-$MAPb_{0.2}Sn_{0.8}I_3$	-	TPBi	Poly-TPD	1.65	2.7	-	885<br>900<br>901<br>909<br>917<br>928<br>943	1.7<br>2.6<br>0.9<br>3.3<br>5<br>2.7<br>0.6	2018	[7]
PbS-ZnO	60	ZnO	PbS	0.6	10	48h	1400	7.9	2019	[8]
CdTe@CdSe	65	ZnO	PDOT:PSS/TFB	1.5	8 mW/cm^2	-	929	1.8	2020	[9]
PbS-Perovskite	45	TPBi	PDOT:PSS	3.7	7.4	-	980	8.1	2020	[10]
i-IDSe-4F	-	PDINO	PDOT:PSS	1.27	43	-	800-1000	0.46	2022	[11]
KI-doped $MAPb_{0.8}Sn_{0.2}I_3$<br>KI-doped $MAPb_{0.6}Sn_{0.4}I_3$<br>KI-doped $MAPb_{0.4}Sn_{0.6}I_3$<br>KI-doped $MAPb_{0.2}Sn_{0.8}I_3$	-	TPBi	Poly-TPD	1.6	1	-	868<br>889<br>897<br>917	9.6<br>7.03<br>3.34<br>0.65	2022	[12]
In(Zn)As–In(Zn)P–GaP–ZnS	75	ZnO:PEIE	Poly-TPD	1.6	8.2		857	4.6	2019	[13]
InAs@ZnSe	43	TPBi	PDOT:PSS / Poly-TPD	2.4	0.2	32h	947	5.5	2022	[14]
In(Zn)As–In(Zn)P–GaP–ZnS	73	ZnO:PVK	Poly-TPD	1.2	20	-	1006	13.3	2022	[15]
$CuInS_2$/ZnS	65	ZnO:PEIE	TFB	1.1	13.3	-	950	8.2	2023	[16]
InAs@ZnSe 7ML shell	70	ZnO:PMMA	Poly-TPD	1.5	12	24h	900	13.3	2024	This work

Reference:

[1] M. Chen *et al.*, “1 micron wavelength photo- and electroluminescence from a conjugated polymer,” *Appl. Phys. Lett.*, vol. 84, no. 18, pp. 3570–3572, May 2004, doi: 10.1063/1.1737064.

[2] R. S. Sanchez, E. Binetti, J. A. Torre, G. Garcia-Belmonte, M. Striccoli, and I. Mora-Sero, “All solution processed low turn-on voltage near infrared LEDs based on core–shell PbS–CdS quantum dots with inverted device structure,” *Nanoscale*, vol. 6, no. 15, pp. 8551–8555, 2014, doi: 10.1039/C4NR01975J.

[3] G. Tregnago, T. T. Steckler, O. Fenwick, M. R. Andersson, and F. Cacialli, “Thia- and selena-diazole containing polymers for near-infrared light-emitting diodes,” *J. Mater. Chem. C*, vol. 3, no. 12, pp. 2792–2797, 2015, doi: 10.1039/C5TC00118H.

[4] W.-L. Hong *et al.*, “Efficient Low-Temperature Solution-Processed Lead-Free Perovskite Infrared Light-Emitting Diodes,” *Adv. Mater.*, vol. 28, no. 36, pp. 8029–8036, Sep. 2016, doi: 10.1002/adma.201601024.

[5] M. L. Lai *et al.*, “Tunable Near-Infrared Luminescence in Tin Halide Perovskite Devices,” *J. Phys. Chem. Lett.*, vol. 7, no. 14, pp. 2653–2658, Jul. 2016, doi: 10.1021/acs.jpclett.6b01047.

[6] H. U. Kim *et al.*, “Substituents engineered deep-red to near-infrared phosphorescence from tris-heteroleptic iridium complexes for solution processable red-NIR organic light-emitting diodes,” *J. Mater. Chem. C*, vol. 6, no. 39, pp. 10640–10658, 2018, doi: 10.1039/C8TC04321C.

[7] W. Qiu *et al.*, “Mixed Lead–Tin Halide Perovskites for Efficient and Wavelength-Tunable Near-Infrared Light-Emitting Diodes,” *Adv. Mater.*, vol. 31, no. 3, p. 1806105, Jan. 2019, doi: 10.1002/adma.201806105.

[8] S. Pradhan *et al.*, “High-efficiency colloidal quantum dot infrared light-emitting diodes via engineering at the supra-nanocrystalline level,” *Nat. Nanotechnol.*, vol. 14, no. 1, pp. 72–79, Jan. 2019, doi: 10.1038/s41565-018-0312-y.

[9] H. Feng *et al.*, “Highly Efficient Near-Infrared Light-Emitting Diodes Based on Chloride Treated CdTe/CdSe Type-II Quantum Dots,” *Front. Chem.*, vol. 8, Apr. 2020, doi: 10.3389/fchem.2020.00266.

[10] L. Gao *et al.*, “Efficient near-infrared light-emitting diodes based on quantum dots in layered perovskite,” *Nat. Photonics*, vol. 14, no. 4, pp. 227–233, Apr. 2020, doi: 10.1038/s41566-019-0577-1.

[11] Y. Xie *et al.*, “Bright short-wavelength infrared organic light-emitting devices,” *Nat. Photonics*, vol. 16, no. 11, pp. 752–761, Nov. 2022, doi: 10.1038/s41566-022-01069-w.

[12] H. Yu, W. Chen, Z. Fang, L. Ding, B. Cao, and Z. Xiao, “Alkalis-doping of mixed tin-lead perovskites for efficient near-infrared light-emitting diodes,” *Sci. Bull.*, vol. 67, no. 1, pp. 54–60, Jan. 2022, doi: 10.1016/j.scib.2021.07.021.

[13] H. Wijaya *et al.*, “Efficient Near-Infrared Light-Emitting Diodes based on In(Zn)As–In(Zn)P–GaP–ZnS Quantum Dots,” *Adv. Funct. Mater.*, vol. 30, no. 4, p. 1906483, Jan. 2020, doi: 10.1002/adfm.201906483.

[14] M. De Franco *et al.*, “Near-Infrared Light-Emitting Diodes Based on RoHS-Compliant InAs/ZnSe Colloidal Quantum Dots,” *ACS Energy Lett.*, pp. 3788–3790, 2022, doi: 10.1021/acsenergylett.2c02070.

[15] X. Zhao, L. J. Lim, S. S. Ang, and Z. Tan, “Efficient Short-Wave Infrared Light-Emitting Diodes Based on Heavy-Metal-Free Quantum Dots,” *Adv. Mater.*, vol. 34, no. 45, p. 2206409, Nov. 2022, doi: 10.1002/adma.202206409.

[16] L. J. Lim, X. Zhao, and Z. Tan, “Non-Toxic CuInS 2 /ZnS Colloidal Quantum Dots for Near-Infrared Light-Emitting Diodes,” *Adv. Mater.*, vol. 35, no. 28, Jul. 2023, doi: 10.1002/adma.202301887.
